# Five-year follow-up of children receiving comprehensive dental care under general anesthesia

**DOI:** 10.1186/1472-6831-14-154

**Published:** 2014-12-15

**Authors:** Nora Savanheimo, Miira M Vehkalahti

**Affiliations:** Department of Oral Public Health, Institute of Dentistry, University of Helsinki, P.O. BOX 41, 00014 Helsinki, Finland; Department of Social Services and Health Care, P.O. BOX 6452, 00099 City of Helsinki, Finland; Department of Oral and Maxillofacial Diseases, Oulu University Hospital, 90220 Oulu, Finland

**Keywords:** Anesthesia, General, Community dentistry, Health services, Outpatient, Preventive dentistry

## Abstract

**Background:**

Dental general anesthesia (DGA) is part of public dental care in Finland, but the intention is to return the patient to routine dental care. The aims of this study were to describe the details of treatments under DGA given to generally healthy children and to explore the outcome of their dental care during a 5-year follow-up, with special focus on preventive care. In particular, we examined the return of the patients to routine dental care, of which, to our knowledge, little is known.

**Methods:**

Our prospective 5-year follow-up of generally healthy children (aged 0–13 years) treated under DGA by the Helsinki Public Dental Service in 2004 was based on official dental and general anesthesia documents. The statistical analyses employed chi-square tests, t-tests, Pearson’s correlation coefficient (r), Fisher’s transformation to test r ≠ 0, and logistic regression modeling.

**Results:**

The most common reason for DGA was uncooperation (82%), followed by dental fear (56%). Filling therapy predominated in the treatments given under anesthesia, and the mean number of treatments per patients was 9.5 (SD = 4.2). Throughout the follow-up, 54% of the patients continued to have co-operation problems and 53% expressed dental fear; 11% of the patients received repeat DGA. The mean follow-up time was 48 (median 52) months. The postoperative review visit was actualized within 1.5 (SD = 0.8) months and the first visit to the home dental clinic of the patients in 12.0 (SD = 11.8) months for the 0–5-year-olds and in 7.2 (SD = 5.9) months for the 6–13-year-olds (p < 0.001). The mean time elapsed to the first need for treatment was 18.5 (SD = 14.1) months. During the follow-up, the mean number of treatments per patient was 5.3 (SD = 4.9); almost all patients (97%) received preventive treatment at one of two visits, but the control of dental fear remained rare.

**Conclusions:**

To return to routine dental care after DGA, most of the generally healthy children in our study still needed special attention due to their uncooperation and dental fear, thus calling for a renewal of practices to treat these patients.

## Background

Dental general anesthesia (DGA) for generally healthy patients is considered a last resort and is not intended for use on a regular basis in dental care. The purpose of DGA is to rehabilitate the patient’s oral health and, if possible, to return the patient to routine dental care.

Similarly to Finland, comprehensive and conservative DGA care for generally healthy children treated as outpatients or in day-stay care has been provided for decades in many countries [1–9]. A different approach whereby DGA is mostly used for extractions has been reported in Australia, England, and South Africa [10–12]. However, there has been a move to more comprehensive care in the United Kingdom and South Africa [12, 13].

After treatment under DGA, children still pose a challenge to the dental service because of their low compliance in attending the services and their high risk of caries relapse. The compliance of generally healthy children in attending the postoperative review visit scheduled two weeks after comprehensive treatment under DGA has been reported to vary between 48–100%, while the compliance in attending the subsequent recalls has tended to decline over time [14–17]. Caries relapse has been found in 37–54% of those returning to 4–6-month recalls [18, 19], and in 53–79% of those returning to recalls within two years [14, 17, 20], a proportion of them again needing treatment under DGA [14, 20].

The aims of this study were to describe the details of treatments under DGA given to generally healthy children and to explore the outcome of their dental care during a 5-year follow-up, with special focus on preventive care. In particular, we examined the return of the patients to routine dental care, of which, to our knowledge, little is known.

## Methods

The data were collected from patient records of all 0–13-year-old generally healthy children treated under DGA in 2004 and of their dental care in the following 5 years. The Department of Social Services and Health Care of City of Helsinki granted ethical permission for this study. The patients were indicated with consecutive numbers for identification in the data analyses.

### Setting

Dental services in Finland are provided in both public and private sectors, although the entire population is entitled to access the Public Dental Service (PDS). Nearly all children use PDS services, which are free of charge for patients below 18 years of age and also include DGA. DGA services in Finland are available in hospitals and in larger public and private dental clinics, with some regional differences in accessibility of the services. Generally, PDS provides DGA for ASA (American Society of Anesthesiologists) grade I–II patients and university hospitals for ASA grade III–IV patients. Regarding PDS, the indications for DGA for children follow the guidelines of the American Academy of Pediatric Dentistry [21].

Each PDS dentist is responsible for treating the child population living in the area of his/her clinic. For the patients, this is their home dental clinic. In the PDS of Helsinki, one of the challenges currently facing dentists is the increasing immigrant population. Of all the immigrants in Finland in 2004, 28% lived in Helsinki, comprising 5% of the 560 000 inhabitants and 17% of the immigrants were aged 0–15 years [22]. DGA in the Helsinki PDS is part of the normal dental service. When PDS dentists confront serious difficulties with a child's dental treatment, they can refer the child to a DGA consultation in a special unit. The Helsinki PDS provided dental services in 2004 for about 140 000 child and adult patients, of whom 322 were treated under DGA.

In the Helsinki PDS, DGA is determined as a comprehensive process, with preventive care being considered as an essential part of it. At a consultation visit preceding DGA, the child and his/her guardian receive rigorous instructions on oral self-care, including appropriate and regular use of fluorides and dietary advice. The DGA treatments are planned and provided by a specialist in pediatrics or by a dentist, who has acquainted him/herself with the DGA. The oral self-care instructions are repeated at the postoperative review visit scheduled about 1 month after DGA, when the child is also familiarized with the dental office and equipment in order to reduce dental fear. If the child is cooperative, professional tooth cleaning and/or topical application of fluoride are performed. Finally, the child and his/her guardian are given instructions on contacting their home dental clinic for a preventive care visit about 2–3 months after the postoperative review visit.

### Patients

**Figure 1:**
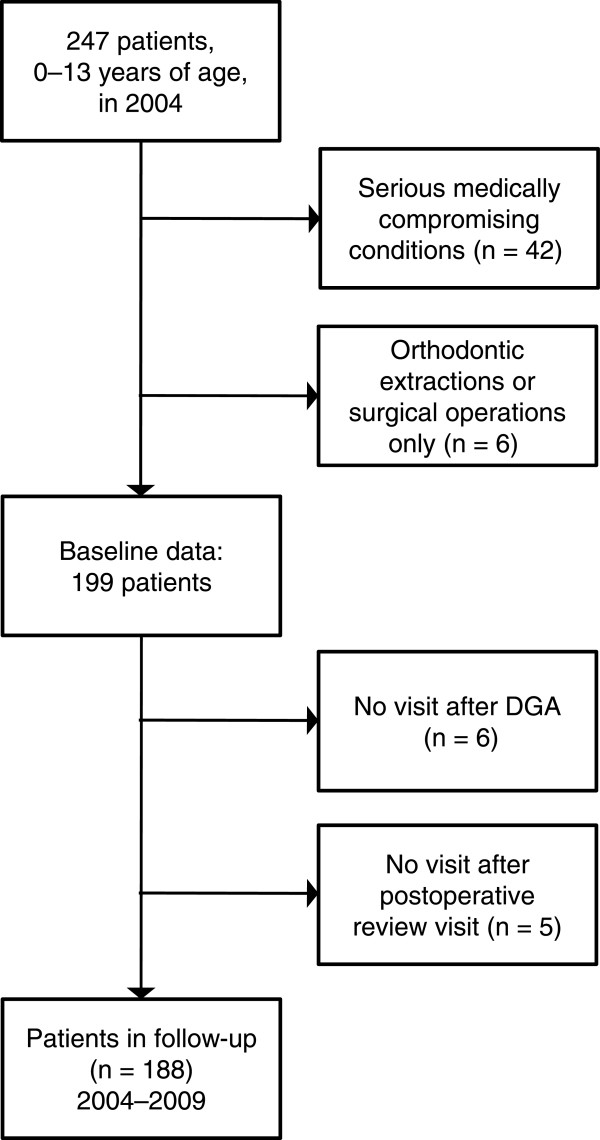
**Flow chart of the dental general anesthesia (DGA) patients.**

The target population (n = 247) comprised all 0–13-year-old patients of the Helsinki PDS receiving DGA in 2004. For this study we included those patients who were generally healthy or had only minor medically compromising conditions such as allergy, atopy, asthma, or a history of recurrent otitis, adenotomy, or tympanostomy. After excluding patients with medically compromising conditions (n = 42) and patients treated with orthodontic extractions or surgical operations (n = 6), the baseline data included 199 patients. Over the 5 years of follow-up, 11 patients dropped out because of moving to another city; the final follow-up data thus included 188 (94%) patients (Figure 1).

### Data collection

The patient’s official dental records together with the documents related to general anesthesia and health history, required from the guardians before the DGA visit, comprised the database. We obtained the information from both electronic and paper documents for final recording into the present data file.

Personal background data included age, gender, whether the individual was an immigrant, and the history of previous conscious sedation (oral midazolam) and/or DGA. Age, to an accuracy of 1 month, was categorized in the analyses as 0–5 and 6–13 years, based on the eruptional stages in the dentition [23]. Immigrant status was defined in terms of nationality or native language.

### DGA visit

The reasons for DGA, noted at the consultation appointment from the PDS dentist’s as a free-text referral, were recorded for this study as extreme uncooperation, extreme dental fear and an excessive need for treatment. Each patient could have multiple reasons.

The numbers of treatments given under DGA were recorded separately for the following services: filling therapy (e.g. glass ionomer, compomer, composite and amalgam restorations, and stainless steel crowns), tooth extractions, endodontics (pulpotomies and root-canal treatments), fissure sealants and prophylaxis (professional tooth cleaning and/or topical application of chlorhexidine or fluoride or minor removal of calculus). For one tooth, multiple treatments were allowed (e.g. endodontics and filling therapy were counted separately).

### Follow-up

The length of the follow-up from the baseline to the end was determined individually from each patient’s DGA visit to his/her last dental visit during the 5-year period following DGA. During the follow-up, the recordings extracted from the patient documents included the time of the postoperative review visit, the time of the first visit to the patient’s home dental clinic, and the time when the first treatment need was diagnosed (e.g. need for filling, endodontics, or extractions) for example because of caries, fractured tooth or filling or periapical infection. We calculated all time intervals to an accuracy of 1 month.

Other data recorded during the follow-up included the number of treatments by type, as at the DGA visit, and the number of dental visits throughout the follow-up. These were recorded separately for the following options, allowing several alternatives for each visit:

- all visits, excluding orthodontic or solely radiographic visits;
- visits in which the patient received preventive treatment, including professional tooth cleaning and/or topical application of chlorhexidine or fluoride, minor removal of calculus, rigorous instructions on oral self-care including appropriate and regular use of fluorides, or dietary advice or fissure sealants made;
- visits with total uncooperation, i.e. a visit in which the patient’s lack of cooperation restricted or hindered dental treatment;
- visits with dental fear, i.e. an appointment in which the patient’s behavior signified dental fear;
- conscious sedation (oral midazolam);
- emergency visits;
- repeat DGA;
- visits to guide the patient in controlling his/her dental fear, e.g. visits to familiarize the patient with the dental office and equipment; and
- missed appointments.

### Statistical analyses

The statistical analyses employed chi-square tests, t-tests, Pearson’s correlation coefficient (r), Fisher’s transformation to test r ≠ 0, and logistic regression modeling. Analyses were performed with the software Survo MM version 3.4.1 [24].

## Results

### Patients

The mean age of the DGA patients treated in 2004 was 6.2 (SD = 2.7) years; half were girls and nearly 60% were under 6 years of age. Table 1 summarizes the DGA patient characteristics. One out of four patients were immigrants, the 0–5-year-olds more often than the older age group (p = 0.03). Previously received conscious sedation (80%) and DGA (23%) were more frequent among the 6–13-year-olds than among the younger patients (p < 0.001).

**Table 1:** **Characteristics (%) of the patients (n = 199) treated under dental general anesthesia (DGA) according to age group**

Characteristics of patients	All n = 199, %	0–5 yr n = 115, %	6–13 yr n = 84, %	***p***
**Gender**				
Boys	56	61	49	0.091
Girls	44	39	51	
**Immigrant**				
Yes	25	30	17	0.026
No	75	70	83	
**Previous sedation**				
Yes	65	54	80	<0.001
No	35	46	20	
**Previous DGA**				
Yes	13	6	23	<0.001
No	87	94	77	
Reasons for DGA*				
**Extreme uncooperation**				
Yes	82	75	93	0.001
No	18	25	7	
**Extreme dental fear**				
Yes	56	45	70	<0.001
No	44	55	30	
**Excessive need for treatment**				
Yes	43	56	27	<0.001
No	57	44	73	

Statistical evaluation using chi-squared tests for differences according to age.

*Reasons for referral to DGA from child’s home dental clinic; multiple reasons recorded. One case missing.

### DGA visit

The vast majority of the patients had uncooperation problems as the reason for DGA and more than half showed extreme dental fear, the 6–13-year-old patients more often than the younger (p = 0.001 and p < 0.001, respectively). An excessive need for treatment was more frequent in the 0–5-year-old patients (p < 0.001).

Almost all of the patients received filling therapy under DGA (Table 2), over two-thirds received extractions, half were given endodontics, and one-fifth fissure sealants. Preventive treatment was rare at the DGA visit. The greatest differences between the age groups were in providing endodontics and fissure sealants. More than half of the 0–5-year-olds and one out of three of the 6–13-year-olds received endodontics (p < 0.001). One out of three of the 6–13-year-olds and one out of 10 of the 0–5-year-olds received fissure sealants (p < 0.001).

**Figure 2:**
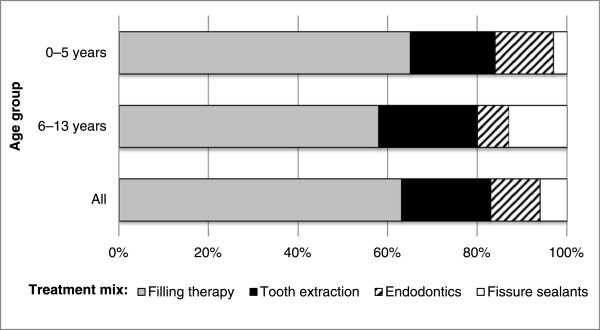
**Treatment mix provided (%) at the dental general anesthesia visit according to age group.**

At the DGA visit, the mean number of treatments (filling therapy, tooth extraction, endodontics, and fissure sealants) per patient was 9.5 (SD = 4.2), being 10.5 (SD = 4.0) for 0–5-year-olds and 8.2 (SD = 4.1) for 6–13-year-olds (p < 0.001). Immigrants had more treatments performed at the DGA visit than had their non-immigrant counterparts (10.7 (SD = 4.6) vs. 9.1 (SD = 4.0); p = 0.03). Among all items of treatment, filling therapy predominated in the treatment mix, followed by extractions (Figure 2).

**Table 2:** **Patients (%) receiving various treatments under dental general anesthesia according to age group (n = 199)**

Treatments received	All n = 199, %	0–5 yr n = 115, %	6–13 yr n = 84, %	***p***
Filling therapy	95	98	92	0.027
Tooth extraction	71	72	70	0.766
Endodontics	48	58	33	<0.001
Fissure sealants	21	10	35	<0.001
Prophylaxis	4	2	7	0.055

Statistical evaluation using chi-squared tests for differences according to age.

### Follow-up

The length of the follow-up averaged 47.6 months (SD = 13.7). Most of the patients could be followed for a lengthy period: the median was 51.5 months, the upper quartile being 56.2 and the lower one 44.8 months.

A postoperative review visit was scheduled for 186 out of 199 patients; 26% missed this appointment. Those 13 patients for whom a postoperative review visit was not scheduled were instructed to contact their home dental clinic within 3 to 6 months. Immigrants attended the postoperative review visit more often than non-immigrants (85% vs. 70%; p = 0.05).

The mean time elapsed from DGA to the postoperative review visit was 1.5 (SD = 0.8) months (Table 3). After DGA, the time interval until the first visit to the patients’ home dental clinic was 12.0 (SD = 11.8) months for the 0–5-year-olds and 7.2 (SD = 5.9) months for the 6–13-year-olds. The mean time that elapsed to the first treatment need after DGA was 18.5 (SD = 14.1) months. The 0–5-year-old patients appeared to remain healthy for longer than the older patients (19.6 vs. 16.9 months), but the difference was statistically non-significant. Those 11% who were treated under repeat DGA received it on average 22.5 (SD = 12.6) months after the initial DGA.

**Figure 3:**
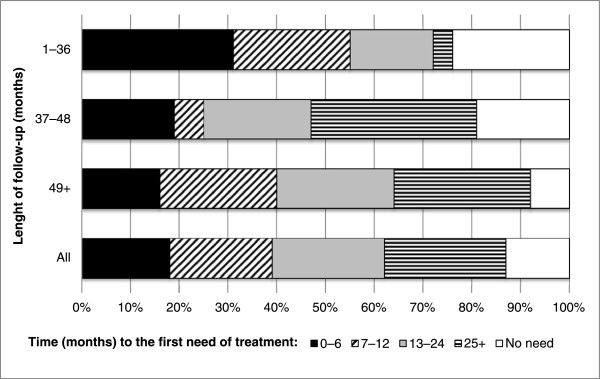
**The first need for treatment following dental general anesthesia according to the length of follow-up.**

Of the patients in follow-up (n = 188), 13% needed no operative treatment during their follow-up period, while 25% were in need of operative treatment not earlier than 2 years after DGA treatment (Figure 3). During the first year after DGA, 39% needed operative treatment, half of them during the first 6 months.

**Table 3:** **Time (months) elapsed to various occasions following dental general anesthesia (DGA) according to age group (n = 188)**

Occasions	n	All Mean (SD)	0–5 yr Mean (SD)	6–13 yr Mean (SD)	***p***
Postoperative review visit	137	1.5 (0.8)	1.4 (0.7)	1.5 (0.8)	0.713
Return to home dental clinic*	188	9.9 (10.0)	12.0 (11.8)	7.2 (5.9)	<0.001
Need for treatment	164	18.5 (14.1)	19.6 (14.7)	16.9 (13.3)	0.225
Repeat DGA	20	22.5 (12.6)	22.1 (9.8)	22.7 (14.6)	0.180

Statistical evaluation using t-tests for differences according to age.

*The responsible clinic, from where the dentist referred the child to DGA treatment.

There was wide variation in the time elapsed from DGA to the first recording of treatment need. To explain this, we fitted logistic regression models to the data, taking as separate outcomes a short (≤12 months) and a long (25+ months) period. None of the explanatory variables tested (the patients’ background information, reasons for DGA, and treatments given under DGA) ended up with statistically significant results.

Table 4 provides the percentages of patients undertaking various types of visits during the follow-up. At their visits, more than half of the patients showed total uncooperation or dental fear, and one out of five received conscious sedation. Visits with total uncooperation were more frequent among boys than girls (62% vs. 45%; p = 0.03). Preventive treatment was given to almost all, emergency treatment to half of the patients and control of dental fear to 13% of patients. Two out of three missed one or more appointments. Of the average number of visits per patient (9.7; SD = 5.6), 4.9 included prevention and 0.2 control of dental fear. There were no differences according to age or immigrant background in the number of the preventive visits, which were more frequent when the follow-up was longer (r = 0.48; p < 0.001).

**Table 4:** **Characteristics of visits (% of patients) following dental general anesthesia (DGA) according to age group (n = 188)**

Characteristics of visits	All n = 188, %	0–5 yr n = 106, %	6–13 yr n = 82, %	***p***
Preventive treatment	97	96	98	0.606
Total uncooperation	54	59	48	0.105
Dental fear	53	56	50	0.441
Conscious sedation	18	23	12	0.065
Emergency treatment	52	55	49	0.419
Repeat DGA	11	8	15	0.118
Control of dental fear	13	15	11	0.410
Missed appointment	65	63	68	0.467

Statistical evaluation using chi-squared tests for differences according to age. Multiple characteristics per patient recorded.

The mean number of treatments during the follow-up was 5.3 (SD = 4.9), including a mean of 4.2 (SD = 4.2) fillings, 0.8 (SD = 1.2) tooth extractions, and 0.3 (SD = 1.1) endodontics, the maximum number being 24 during 5 years. There were no differences between the age groups.

## Discussion

The main reason for the use of DGA in generally healthy children in our study was uncooperation problems, followed by dental fear and excessive need for treatment. Even though the initial treatment need at the DGA visit was extensive, the treatment need during the follow-up was at the same level as in the general population. Most of the patients could be treated in routine dental care after DGA, but many difficulties persisted in their dental care. During the follow-up, almost all patients (97%) received preventive treatment at one out of two visits. Most of the patients could be followed for a lengthy period: three out of four for about 45 months.

A limitation in our study might be our patient record-based data, as in many other studies [14, 16, 17, 20]. In general, the records can be taken as reliable, because there are statutory rules for the recording and treatment codes used in patient records in Finland. In addition, part of the salary of dentists in the public sector is based on treatment fees calculated according to the treatment codes in patient records. However, dentists may vary in their recording practices, in particular the coding of prevention and the use of familiarization, for which treatment fees are not paid, so these activities may thus have been underrepresented in our data. Since every city in Finland has its own patient record system, those patients who moved to another city during the follow-up were lost from our data. Similarly, if a patient selected private sector treatment, these visits would not have been observed in our study.

Preventive treatment and encouragement to follow oral self-care recommendations should be repeated regularly and frequently enough to obtain the most favorable outcomes [25]. At least short-term improvement in compliance with oral self-care recommendations after DGA has been reported by parents [26–28], but to maintain compliance with the instructions given, parents would like more training and support with their children’s oral self-care [29]. In Finland, this support is offered by PDS for all children, since they are regularly called to dental check-ups at their home dental clinic according to their individual needs.

Preventive care is an essential part of the DGA process in the Helsinki PDS. All the children in our study received preventive care at the consultation appointment, and three out of four of these children received their next preventive care at the postoperative review visit. Later, preventive treatment was given to almost all of our follow-up patients at every second dental visit, but for high-risk patients, this may not have been enough. A report from the USA [14] found no significant effect of the frequency of recall visits on the development of new carious lesions in early childhood caries (ECC) when the recall interval after DGA was 6–9 months, which was also the approximate actualized preventive care interval for children in our study. Nevertheless, preventive care was an essential part of the dental care in our follow-up, since the longer the follow-up was, the more preventive care visits the children attended.

Compliance in attending the postoperative review visit during the time recommended in our study was average compared to other studies [14–17]. This may have been influenced the fact that every child in Finland has a home dental clinic, which has been noted as an important factor in improving the attendance at postoperative visits after DGA [30]. As described earlier, the compliance in attending recalls tends to cease over time. This was also observed in our results; even though the postoperative review visit was performed in the recommended time, the first visit to the child’s home dental clinic was typically later than advised, thus leading to disruption of the scheduled intensified preventive care process.

The children in our study were part of the normal population. Many previous studies concerning DGA and subsequent follow-up have focused on children with ECC, often with a lower socio-economic status. An exception, however, was a study from Lithuania, in which the parents of the DGA children had a higher educational level than the general population [31]. Patient records in Finland include no information on the child’s family background, except for the native language of the child or the parents, providing evidence of their possibly immigrancy. While this is likely to indicate cultural differences, the immigrants in our study, who comprised one-fourth of the children treated under DGA, only differed in having a greater number of treatments performed at the DGA visit, in line with a report on Danish children [8]. In addition, our immigrant patients showed better compliance in attending the postoperative review visits than non-immigrants.

Four out of five children in our study showed extreme uncooperation and over half expressed extreme dental fear as the reason for DGA. The children also continued to have difficulties in their dental care during the follow-up. Even though only one out of nine children required repeat DGA during the follow-up, over half of the patients presented with total uncooperation and/or dental fear. In addition, two out of three patients missed appointments, also indicating ongoing behavior management problems as the reason for avoiding dental care.

To guide the child back to normal dental care after DGA, dental fear needs to be managed, since it is strongly associated with behavior management problems [32, 33]. One gentle way to treat dental fear is to familiarize the child with the dental office and equipment, but only one out of eight patients in our study received this type of guidance after the postoperative review visit. To improve the compliance of uncooperative patients in the long term, familiarization should be strongly prioritized. The first visit to the child’s home dental clinic within the recommended time would have served as familiarization, but the children and their parents in our study attended this appointment later than recommended.

Although the individual variation was wide, the children’s first treatment need occurred on average 18.5 months after DGA. This is in line with a report from the USA [14], although in that study only new carious lesions were recorded. Another study from USA reported notable success for ECC children, of whom only 26% developed new caries within 3 years after DGA [16]. Unfortunately, only 5% of the initially treated patients completed the follow-up in that study, thus reducing the generalizability of the findings.

Compared to earlier studies, the follow-up of the patients in our study was much longer, with three out of four being followed for 45 months or longer. This may partly be explained by the fact that PDS clinics and the DGA unit have a joint patient record system. Thus, following the dental care of these patients is not only dependent on their attending specific appointments. Since DGA in the PDS in Finland is free of charge for patients below 18 years of age, the costs of the service and subsequent dental care have no influence on attendance of the dental service.

## Conclusions

Our study demonstrated that after comprehensive dental care under DGA, most of the generally healthy children can be treated in routine dental care, but they still require special attention due to their uncooperation and dental fear also in order to reduce the number of missed appointments. Extensive and comprehensive DGA treatment appears to keep the need for further treatment at a similar level to that in the general population. After DGA, familiarization with dental care must be strongly prioritized.
